# Functionalization and Surface Modifications of Bioactive Glasses (BGs): Tailoring of the Biological Response Working on the Outermost Surface Layer

**DOI:** 10.3390/ma12223696

**Published:** 2019-11-09

**Authors:** Saeid Kargozar, Farzad Kermani, Sahar Mollazadeh Beidokhti, Sepideh Hamzehlou, Enrica Verné, Sara Ferraris, Francesco Baino

**Affiliations:** 1 Tissue Engineering Research Group (TERG), Department of Anatomy and Cell Biology, School of Medicine, Mashhad University of Medical Sciences, Mashhad 917794-8564, Iran; 2 Department of Materials Engineering, Faculty of Engineering, Ferdowsi University of Mashhad (FUM), Azadi Sq., Mashhad 917794-8564, Iran; farzadkermani73@gmail.com (F.K.); mollazadeh.b@um.ac.ir (S.M.B.); 3 Department of Medical Genetics, School of Medicine, Tehran University of Medical Sciences, Tehran 14155-6447, Iran; 4 Institute of Materials Physics and Engineering, Applied Science and Technology Department, Politecnico di Torino, Corso Duca degli Abruzzi 24, 10129 Torino, Italy; enrica.verne@polito.it (E.V.); sara.ferraris@polito.it (S.F.)

**Keywords:** bioactive glasses, surface modifications, functionalization, bioactivity, bone tissue engineering

## Abstract

Bioactive glasses (BGs) are routinely being used as potent materials for hard and soft tissue engineering applications; however, improving their biological activities through surface functionalization and modification has been underestimated so far. The surface characteristics of BGs are key factors in determining the success of any implanted BG-based material in vivo since they regulate the affinity and binding of different biological macromolecules and thereby the interactions between cells and the implant. Therefore, a number of strategies using chemical agents (e.g., glutaraldehyde, silanes) and physical methods (e.g., laser treatment) have been evaluated and applied to design properly, tailor, and improve the surface properties of BGs. All these approaches aim at enhancing the biological activities of BGs, including the induction of cell proliferation and subsequent osteogenesis, as well as the inhibition of bacterial growth and adhesion, thereby reducing infection. In this study, we present an overview of the currently used approaches of surface functionalization and modifications of BGs, along with discussing the biological outputs induced by these changes.

## 1. Introduction 

In the field of biomaterials, bioactive glasses (BGs) have been extensively used for hard and soft tissue engineering applications from tissue healing to cancer therapy [1,2,3,4,5,6,7]. It has been well documented that BGs can promote the tissue healing process by locally releasing specific metal ions at therapeutic concentrations [8,9,10]. However, the ability to bond to the living tissues, i.e., bioactivity, is recognized as the most prominent feature of BGs as compared to other types of biomaterials when they are implanted in the human body. The formation of a hydroxycarbonate apatite (HCA) layer on the glass surface after contact (within minutes to hours) with biological fluids (e.g., plasma) is regarded as a prerequisite for bonding to the bone and collagenous tissues. The formation of an HCA layer on the surface of BGs was also shown to occur in vitro upon soaking in a simulated body fluid (SBF) mimicking the inorganic composition of human plasma [11]. Therefore, immersion studies in SBF have been adopted by the scientific community as a simple test to assess the HCA-forming capability of biomaterials [12]. However, it was pointed out that bioactivity measurement using SBF may lead to false positive and false negative results in some cases [13]; hence, over the last years, there has been an increasing effort in refining the SBF-based testing method [14,15,16].

**Figure 1:**
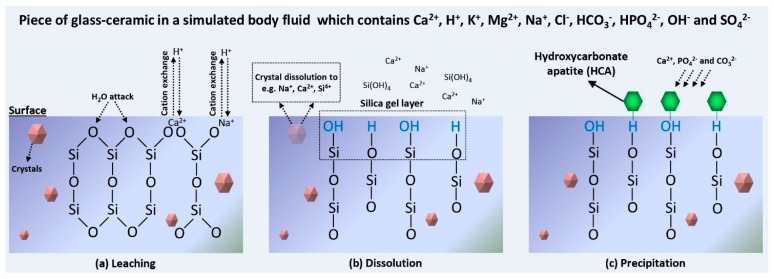
A series of surface chemical reactions including (**a**) leaching, (**b**) dissolution, and (**c**) precipitation occurs after immersion of BGs and glass-ceramics in biological fluids (e.g., plasma and SBF) to form a hydroxycarbonate apatite (HCA) layer on their surface within minutes to hours.

HCA forms through a series of chemical reactions in the biological environment [17] (Figure 1). Traditionally, the glasses with up to ~53 mol.% SiO_2_ in the composition of Na_2_O-CaO-P_2_O_5_-SiO_2_ are recognized as materials with the ability to bond to both hard (e.g., bone) and soft (e.g., skin) tissues, while those with 53 to 60 mol.% SiO_2_ can only bond to the bone. From a general viewpoint, melt-derived silicate glasses with less than 60 mol.% SiO_2_ are identified as bioactive materials [18]. 

Many researchers have taken benefit from the bioactivity of BGs to improve the surface characteristics of bioinert materials like inert bioceramics (e.g., zirconia and alumina) [19]. Metallic implants have also been coated with BGs by using various methods, such as plasma-sprayed deposition technique, electrophoretic deposition, sol-gel process, and magnetron sputtering [20,21,22]. 

Methods aimed at functionalizing or somehow modifying the surface of BG powders or scaffolds have also been proposed to improve the glass performance further, and obtain more desirable outputs regarding the biological applications [23]. As an illustration, the enhanced binding of proteins on the BGs was observed after functionalizing their surface with glutaraldehyde (GA) [24]. Moreover, improvement of the glass biocompatibility was also documented as an additional result of the surface modification of the glasses [25]. 

Generally, the surface of BGs can be modified by physical (e.g., change in surface topography) and chemical/biochemical (e.g., adsorption of molecules by atomic layer deposition, covalent grafting of biomolecules/drugs, etc.) approaches. Surface reactivity of bioactive glasses can be successfully exploited in the functionalization strategies, and the typical properties of BGs (e.g., ion release, bioactivity) can be maintained and combined with new customized functional features (e.g., antibacterial, antioxidant, anticancer properties, etc.) [26,27]. Moreover, surface modification of bioactive glasses can be intended both for the improvement of bioactivity/biological response, as well as for the compatibilization of glass particles with other phases [28]. Most of the methods used for surface modification of BGs are usually simple, straightforward and relatively inexpensive. 

Apart from “conventional” melt-derived BGs, it has been shown that surface modification can be useful for improving the biological functions of mesoporous BGs (MBGs), too. For instance, the improvement in the amount of drug-loaded into and released from MBGs has been previously documented as a result of surface modification [29].

In this review study, we describe the importance of surface modifications of BGs and show their effects on biological activities in the living systems. Although there is a large number of studies on surface modification of other biomaterials, such as calcium phosphates, little research is available on functionalizing different formulations of BGs. Therefore, we aim to bridge this gap and highlight the importance of this issue in the field of glass science in order to promote discussion and stimulate further research. 

## 2. Mechanisms of Surface Modification of BGs 

It has been previously well studied that surface properties of biomaterials are very important factors controlling the fate of their implantation in vivo. Physical properties of the surface, including topography, particle size (in the case of particulate systems), porosity, pore size, and charge can dictate the interactions between biological components (e.g., proteins) and biomaterials [30]. These aspects, which essentially involve the tailoring of surface micro- and nano-roughness to elicit a favorable biological response (e.g., improved cell adhesion or tissue on-growth), have been intensively investigated for metallic biomaterials [31]. Surface nano-texturing seems to be less important in the case of bioactive glasses that are inherently reactive upon contact with biological fluids and tend to be coated by a surface HCA layer. It was demonstrated that higher the surface nano-porosity, higher the specific surface area and, hence, higher the apatite-forming kinetics of bioactive glasses (highly-porous sol-gel vs. melt-derived materials) [32,33,34,35]; however, fast bioactive reactions were observed both in vitro and in vivo (actual bonding to hard and soft tissues) also when non-porous biomedical glasses are used [36,37,38,39].

Strategies of chemical surface modification using coupling agents or pH changes are more suitable and effective in the case of BGs. Other approaches of surface modification involve the deposition of coatings, the use of radiations and the development of core-shell systems. Table 1 summarizes the results of previous works dealing with the surface modification of BGs by different methods, which are discussed in the following sections [26].

### 2.1. Surface Modification by Chemical Agents

Among the various substances used for surface modifications of biomaterials, surfactants are considered as the most powerful agents with satisfactory outcomes [51,52,53]. Moreover, the functionalizing process using surfactants is simple, straightforward, and without the high costs. 

Several functional groups (e.g., NH_2_ or COOH) have been introduced on the surface of BGs in order to act as active sites for specific properties or further molecular grafting. The most commonly-used one is the amino group (NH_2_), introduced on the surface of BGs by means of silanization with (3-aminopropyl) triethoxysilane (APTS, C_9_H_23_NO_3_Si). A summary of these surface modifications has been discussed by Ferraris and Verné in Reference [26] and is briefly summarized here in Table 2.

**Figure 2:**
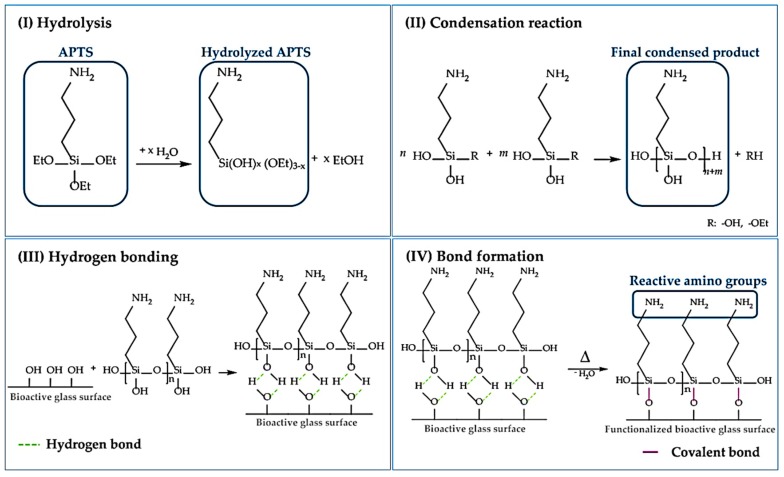
Schematic representation of different steps of surface functionalization of 45S5 glass-based scaffolds by using APTS. Adapted from Reference [43].

The role of APTS in the surface functionalization of BGs is shown in Figure 2. APTS was reported by Verné et al. as a proper agent for covalently bonding bone morphogenetic proteins (e.g., BMP-2) to the surface of BGs, like CEL2 (molar composition—45% SiO_2_, 3% P_2_O_5_, 3%, 26% CaO, 7% MgO, 15% Na_2_O, 4% K_2_O) [40]. APTS is considered as a common silane agent containing amino groups, which could promote the formation of spherical HCA agglomerates without decreasing the bioactivity of the glasses [56]. In an aqueous environment, the hydrolysis of APTS leads to the formation of silanol groups, which could react with hydroxyl groups onto surfaces [66]. Figure 2 shows the different steps involved in the surface modification of 45S5 Bioglass^®^-based scaffolds through the treatment with APTS. As shown, the surface modification could be divided into four steps including (I): Hydrolysis, (II) condensation reaction, (III) hydrogen bonding, and (IV) bond formation [43].

**Figure 3:**
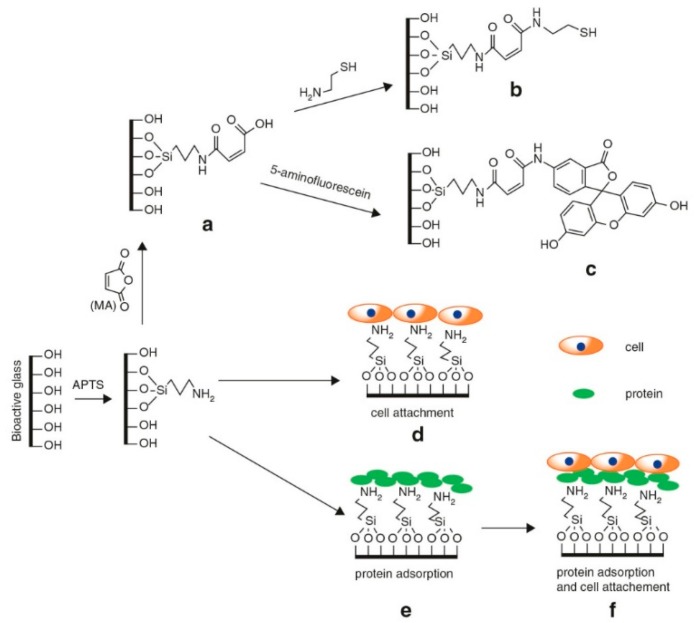
Schematic representation of procedure of (**a**) the preparation of APTS-BG-maleic acid (MA); (**b**) synthesis of the APTS BG-MA and cysteamine conjugate; (**c**) the APTS-BG-MA and 5-aminoflorescein conjugate; (**d**,**f**) models for cell binding; and protein adsorption (**e**,**f**). With some modifications from ref. [70].

Apart from promoting bioactivity, APTS has been used in several studies to improve other properties of BGs, such as cytocompatibility. As an illustration, Magyari and coworkers assessed and showed blood compatibility of SiO_2_–CaO–P_2_O_5_ glasses after surface treatment with APTS [67]. Moreover, Chen et al. could successfully modify the surface of 58S sol-gel derived BG (60 mol.% SiO_2_, 36 mol.% CaO, 4 mol.% P_2_O_5_) using APTS in order to improve the cytocompatibility of samples [68]. Their study was based on the fact that NH_2_-terminated materials (e.g., APTS functionalized BGs) show better cell viability and proliferation in comparison to –OH, –COOH and –CH_3_ terminated substances (Figure 3) [25,69]. 

In addition to silica-based bioactive glasses, APTS was successfully grafted to phosphate and borate glasses, too [71,72], thus, opening the opportunity for the surface functionalization of these materials.

Several techniques have been successfully employed for the study of the silanization process on BGs. SEM and EDS analyses can be used for the determination of morphological (surface layer formation) and chemical composition changes (variation of Si, N surface content) attributable to APTS presence [57], XPS analyses are also used for the detection of silane molecules on the glass surface, as well as of the typical functional groups of silanes [40,64,71,72]. The calculation of atomic ratios between the characteristic elements of APTS molecules (e.g., N) and the substrate ones can give an indication of the silane layer thickness. Similarly, the comparison between the chemical composition results obtained with EDS and XPS, which have different penetration depth (1 μm vs. few nanometers, respectively) can give indications on the thickness of the organic layer. Moreover, FTIR spectroscopy has been employed for the investigation of APTS functional groups on the glass surface (mainly powder) with good results [57,73]. Finally, contact angle measurement has been shown as a fast and effective method for the determination of silane molecule presence on the glass surface [40,64,71,72]. In fact, BGs are highly hydrophilic, due to the presence of OH groups on their surface, whereas APTS is a hydrophobic organic molecule. APTS presence on the glass surface significantly changes the wettability of the material, and this variation can be easily detected.

Recently, the use of zeta potential electrokinetic measurement has been indicated as a promising technique for the determination of the presence of a silane molecule on the surface of BGs [72]. The technique detects charge variation at the glass surface in the function of pH and can be successfully used for the monitoring of surface changes upon functionalization (with different kinds of molecules). The high reactivity of BGs upon contact with aqueous solutions (e.g., the electrolyte used for the measurements) can induce some artifacts which should be taken into account.

Glutaraldehyde (GA) is another agent used for surface modification of BGs, which could facilitate protein attachment on the glass surface and improve the protein binding ability [45,73,74,75]. GA is usually anchored to the surface of BGs by using APTS as an intermediate. In this route, GA forms an imine bond with the NH_2_ groups of the silane, as suggested by Leivo et al. [76]; however, it should be noted that this agent is a cytotoxic substance if not bound to the surface of samples. Gruian et al. in 2012 evaluated the effect of GA as a coupling agent for protein adsorption on a sol-gel BG (45SiO_2_-24.5Na_2_O-24.5CaO-6P_2_O_5_ mol.%) and showed that the modified samples could provide a better protein adherence [24]. The same research group in 2013 functionalized another sol-gel BG (56SiO_2_·(40 − x)CaO·4P_2_O_5_·xAg_2_O system, with x = 0, 2, and 8 mol.%) with GA in order to evaluate its hemoglobin affinity [77]. The authors showed that the surface modification using GA results in an improvement in the stability of protein attachment and induce polymerization of hemoglobin molecules.

Tetraethoxysilane (TEOS, C_8_H_20_O), as the source of silicate, is one of the most widely used compounds in the synthesis process of BGs. TEOS can also be used as a surface-modifying agent for BG surface through the formation of a silica layer with a negative charge (i.e., Si–O^−^) [46]. Lusvardi et al. manipulated the surface of Ga-doped 45S5 Bioglass^®^ by using TEOS and APTS separately as surface-modifying agents [46]. They showed that the immobilization of APTS on the surface of the glass was less than TEOS. Accordingly, the thickness of the silica layer formed around the glass surface was also higher in the case of TEOS. 

**Figure 4:**
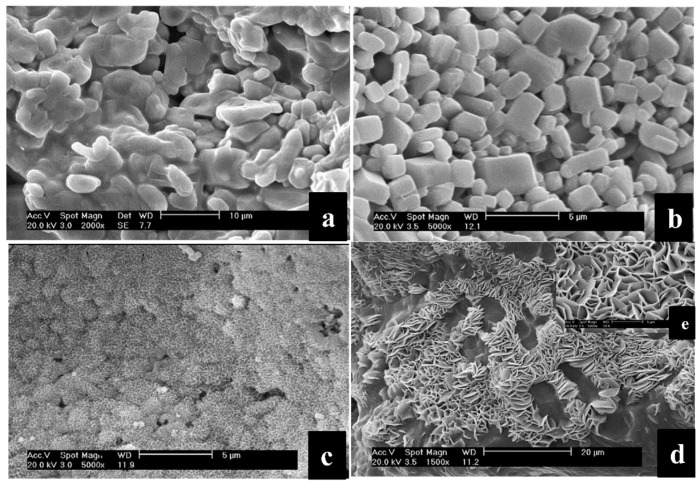
SEM micrographs of (**a**) glass-ceramics of the CaO–P_2_O_5_–Na_2_O–SrO–ZnO system sintered at 650 °C for 4 h, (**b**) the samples after treatment by HCl solution at pH 1.0 for 2.5 min, (**c**) pH 3.0 for 2.5 min, (**d**) pH 10.0 for 2.5 min, and (**e**) pH 10.0 for 10.0 min. Reproduced with permission from Reference [49].

The solubility of BGs was influenced by several factors, such as the composition and structure of glass, presence of crystalline phase embedded in a glassy matrix (glass-ceramic materials), and local pH of the environment. On this matter, Li et al. evaluated the effect of different pH values on the solubility and surface modification of phosphate sol-gel bioactive glass-ceramics in the CaO–P_2_O_5_–Na_2_O–SrO–ZnO system [49]. They immersed the synthesized samples in acidic (HCl, HF) or alkaline (ammonia-based) solutions at various pH and observed: (1) The residual glass matrix on the surface without any crystalline phase for samples soaked in solution at pH 1.0; (2) web-like layer corresponding to CaP_2_O_6_ covering the entire surface of the sample soaked in solution at pH 3.0; (3) partial dissolution of the glass matrix, as well as precipitation of a new phase Ca_4_P_6_O_19_ forming a petaline layer in solution at pH 10.0 (Figure 4). The authors introduced this approach as a simple chemical treatment creating the surface morphology modifications and phase composition. They suggested that the higher level of surface roughness in the newly-formed layer could be beneficial for cell adhesion, due to the increased area available for the cell-implant interfacial bonding.

### 2.2. Physico-Chemical Techniques 

**Figure 5:**
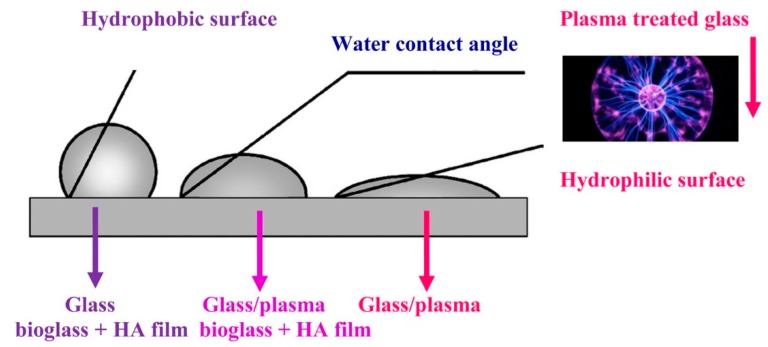
The schematic illustration of the surface wettability changes before and after plasma treatment. Reproduced with permission from Reference [78].

The approaches belonging to this class include the use of plasma, coating procedures or ion-exchange strategies for modifying the surface composition of BGs. Deposition of polymeric coatings combined with plasma activation of BG surface was proposed as a valuable strategy to properly tailor the implant surface characteristics. It has been well understood that the formation of polymeric materials can happen under the influence of ionized gas (plasma) [47]; hence, plasma polymerization is recognized as a rapid and clean method for the modification of glasses and BGs in a wet process [47]. For example, Wiacek et al. increased the surface wetting and adhesion properties of optical glass plates covered with a BG layer in hyaluronic acid (HA), alginate (AL) or mixed solution by using air plasma treatment in the low-temperature plasma system (see Figure 5) [78]. They took advantage of this approach to return hydrophilicity of glass surface, which usually decreases after attachment of BG in the polysaccharide solution on the original glass plates. The authors stated that all changes of glass surface were obviously determined by the kind of probe liquid and concentration of BG in the polysaccharide solution. 

Larrañaga et al. reported a successful surface treatment of 45S5 Bioglass^®^ particles by using plasma polymerization of acrylic acid to improve the thermal stability of composites made of BG and poly(L-lactide) (PLLA), poly(ε-caprolactone) (PCL) and poly(L-lactide/ε-caprolactone) (PLCL) [47]. This approach resulted in preventing the degradation reaction between the Si-O^−^ and C=O groups present on the surface of the glass and polymeric backbone, respectively. Thermal stability was improved accordingly as the onset degradation temperature showed a significant increase in 45S5 Bioglass^®^-filled composites compared to glass-free polymeric counterparts.

Glass-ceramic scaffolds coated with melanin, a natural-derived polymer (pigment) extracted from a cuttlefish (Sepia officinalis), were developed by Araujo et al. [79]. The melanin-coated scaffolds were obtained applying a vacuum-assisted dip-coating method and exhibited improved properties in comparison with the uncoated ones, such as faster bioactivity, enhanced mechanical strength and local drug delivery ability, maintaining unaltered the porosity of the glass-ceramic scaffold.

**Figure 6:**
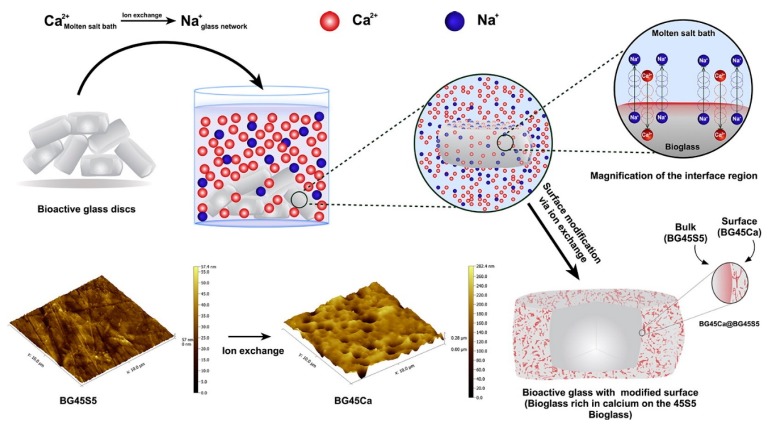
Schematic image of surface modification of 45S5 bioglass by ion exchange technique via immersion of the samples in a molten salt bath. Reproduced with permission from ref [63].

The formation of core-shell based system is another important technique applied for developing glasses and glass-ceramics having desirable surface characteristics [80]. Having a more reactive shell at the surface of BGs could be beneficial to further accelerate HCA layer formation and, hence, the implant attachment to the tissue. Moreover, this reactive shell could increase the roughness of the surface, which is an appropriate event for favoring HCA layer formation [48]. Figure 6 shows the topography changes created by the core-shell based system developed by Lopes et al. [48]. They modified the surface of melt-derived 45S5 Bioglass^®^ discs by a Ca^2+^-Na^+^ ion-exchange process: Specifically, the glass was immersed in a mixture of molten salts (Ca(NO_3_)_2_ and NaNO_3_ with molar ratio of 70:30) at 480 °C for different time frames (0 to 60 min), and thus, the glass composition could be finely tuned, due to Ca^2+^-for-Na^+^ replacement. The authors stated that selectively changing the chemical composition of the surface layer of the parent BG may create new and more reactive glasses in a shell that surrounds the unchanged core (core-shell type system). The change introduced by the authors was illustrated by the following reaction: Ca^2+^_molten salt bath_ + Na^+^_glass_ ←→ Ca^2+^_glass_ + Na^+^_molten salt bath_ (Figure 6). They showed that, via this core-shell system, the formation of the silica gel layer was promoted, and the HCA layer formation accelerated, thereby having a positive impact on bioactivity.

Ion exchange, both in molten salts and in aqueous solutions [81,82,83], has also been used for silver surface enrichment of bioactive glasses and glass-ceramics in order to impart antibacterial properties. This route allows confining silver in the outermost surface layer avoiding the use of huge amounts of this metal and consequently reducing costs and cytotoxicity risks. Moreover, the only fraction of silver useful for antibacterial purposes is the one released from the surface: Therefore, by this process, only the effective silver is introduced in the material. Finally, the ion-exchange process can be applied to glass and glass-ceramic components of complex shapes, as well as on coatings and devices [84,85].

### 2.3. Radiation-Based Methods

A relatively underexplored family of physical strategies to modify the surface of BGs involves the use of radiation with various wavelengths. BG dissolution was shown to increase after gamma irradiation that led to the generation of non-bridging oxygens in the glass network, with significant effects on bioactivity and biocompatibility [86]. It has been shown that gamma irradiation (25 kGy) of BG has an impact on the cell biological response, resulting in enhanced proliferation of normal fibroblast cells [86]. 

Micrometric and nanometric texturing were also performed by applying an infrared laser beam on the surface of melt-derived 45S5 Bioglass^®^ to improve the bioactive properties [87]. Laser texturing is more popular to obtain microrough patterns on titanium surfaces [88], which are known to promote osteoblast attachment and proliferation onto orthopedic implants [31].

## 3. Biological Improvements Carried by Surface Functionalization

Surface treatment of BGs is an effective approach to boosting the biological properties of BGs, including increased cell proliferation, osteogenesis, anticancer and antibacterial activities. Surface functionalization was recently applied to MBGs in the attempt of combining the excellent apatite-forming ability of these nano-textured glasses with additional extra-functionalities of biological interest. For example, surface functionalization of MBG-based scaffolds with the aim of improving the attachment, proliferation, and differentiation of human bone marrow-derived mesenchymal stem cells (hBMSCs) has been reported by Zhao et al. [89]. They functionalized the surface of MBG scaffolds by using thiol (SH) and amino (NH_2_) groups to form thiol-functionalized MBG (SH-MBG) and amino-functionalized MBG (NH_2_-MBG) scaffolds. The functionalization process had no adverse effects on the textural properties of the glasses, including hierarchical pore architecture (from the meso- to the macro-scale) and total porosity. Moreover, SH-MBG and NH_2_-MBG scaffolds showed similar apatite mineralization ability, as well as cytocompatibility in comparison to the untreated MBG scaffolds. As an important outcome, the SH-MBG and NH_2_-MBG scaffold significantly improved the attachment, proliferation, and differentiation of hBMSCs, confirming the beneficial effect of this kind of surface functionalization for bone tissue engineering applications.

In 2018, Hum and Boccaccini could successfully decorate the surface of 45S5 Bioglass^®^-based porous scaffolds using collagen to improve the biological activity of the samples [43]. They cleaned the glass surface to expose reactive –OH groups, and then functionalized the samples using APTS. At the next step, the functionalized glasses were coated by immersion in collagen solution and finally stabilized by cross-linking with 1-ethyl-3-(3-dimethylaminopropyl)carbodiimide (EDC) and N-hydroxysuccinimide (NHS). Their results revealed that coating the BG with collagen could result in a significant increase of compressive strength (up to five times) without any adverse effects on the scaffold macroporosity. Moreover, the results obtained from cell viability assay showed an improvement in the proliferation of MG-63 cell line when cultured with the cross-linked and coated glasses in comparison to other groups (uncoated and uncross-linked samples).

Osseointegration, which is a key factor in determining the success of bone implants, is also affected by the surface texture that can be produced by laser ablation. On this matter, Shaikh et al. [87] evaluated the effects of the irradiation of a nanosecond laser beam on structural, surface morphology and bioactive property of melt-quenched 45S5 Bioglass^®^. This irradiation caused the formation of porous microstructures (pore size of 50 nm to 2 μm) onto the glass and resulted in a significant improvement in the formation of an HCA layer on the sample surface after incubation in SBF. The authors claimed that this improvement could be effective to promote osseointegration and, thereby, accelerate bone healing.

**Figure 7:**
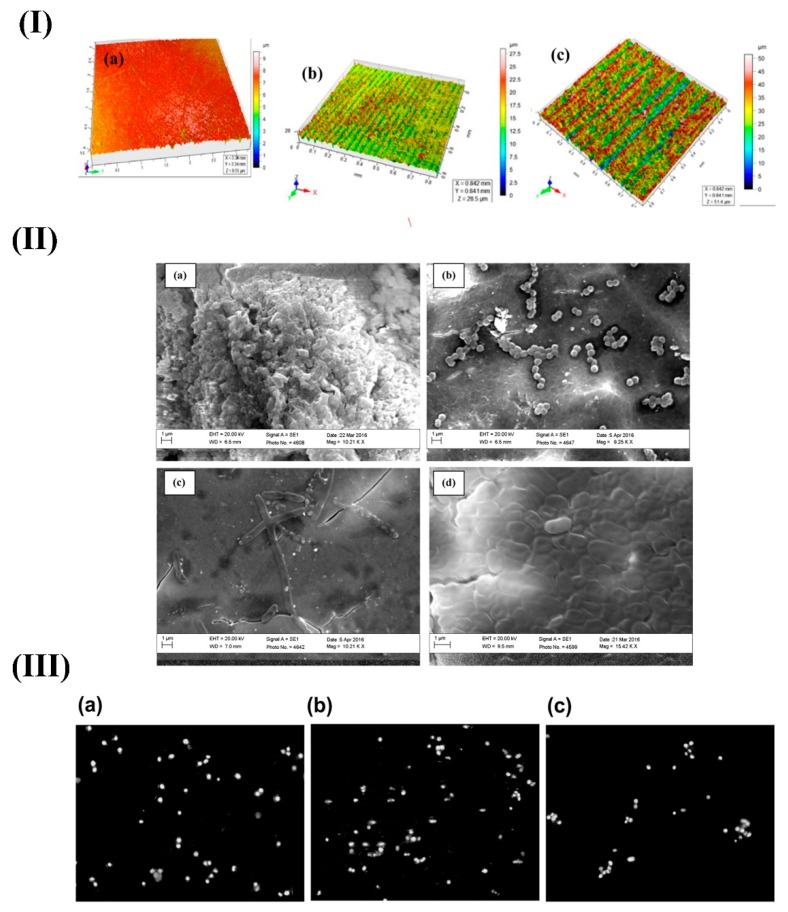
The biological outcomes of surface treatment of BGs by using the laser. (**I**) optical profilometer image of untreated BG (**a**) (sample1); laser-treated at fluence 1.0 J/cm^2^ and sample scanning speed of 35 μm/s (**b**) (sample2); and 15 μm/s (**c**) (sample3). (**II**) SEM micrographs of sample 3 exhibiting no adherence of *S. aureus* (**a**); micrographs showing the attachment of *S. aureus* (**b**), *P. aeruginosa* (**c**), and (**d**) *E. coli* on the surface of untreated BGs. (**III**) The proliferation of INT407 cell line on untreated BGs, the sample3 and (**c**) Petri dish. Reproduced with permission from Reference [90].

Besides the improvement of bonding to the bone, another medical application of surface modification of BGs is enhancing their antibacterial properties, which could prevent the formation of biofilm on their surface [19,79,80,81,82,83]. On this matter, Verné and co-workers [82] developed a simple method of surface modification by ion exchange to impart antibacterial properties to bioactive glasses and glass-ceramics. Shaikh et al. modified the surface of the 45S5 Bioglass^®^ using a femtosecond laser and investigated its effect on the prevention of adhesion of three species of bacteria (*Staphylococcus aureus (S. aureus*), *Pseudomonas aeruginosa* (*P. aeruginosa*) and *Escherichia coli* (*E. coli*)) [90]. The authors treated the surface topography of melt-quenched BG samples using titanium: Sapphire femtosecond pulsed laser by direct laser writing technique. The reason for selecting ultra-short pulsed laser-based surface modification was its advantages, including being a single step, chemical-free process and providing surface treatment localized in both time and space. The obtained results suggested the potential for an enhanced bio-integration of the samples treated with femtosecond laser, due to the superior and faster growth of crystalline HCA-like layer as compared with those treated by the nanosecond laser beam. Moreover, increased roughness and wettability were observed on the glass surfaces after treatment with laser, thereby affecting the surface area of the samples. Since the interactions between bacterial cells and BG surface are primarily determined by micrometric and sub-micrometric levels of surface roughness, the maximum inhibition of bacterial attachment was actually observed in the samples with the highest surface roughness. As another promising outcome, the cytocompatibility of the samples did not reduce after treatment by the laser, further confirming the suitability of this method in imparting desirable characteristics to BG-based bio-implants and devices (see Figure 7).

In another study, Zheng et al. prepared monodispersed lysozyme (LY)-functionalized BG nanoparticles for antibacterial and anticancer strategies [91]. They took benefit from a simple electrostatic interaction routine to functionalize the BGs by LY molecules. Although the LY-functionalized samples showed bioactivity after immersion in SBF for seven days, the formation of an HCA layer was retarded in comparison to the non-functionalized BG nanoparticles. Antibacterial effects of the LY-BGs were confirmed as the samples at a concentration of 1 mg/mL could kill more than 90% of Gram-positive *Bacillus subtilis* (*B. subtilis*) at 24 h post-incubation. Moreover, the LY-BGs were cytotoxic materials for the human hepatocellular carcinoma (HepG2) cell line, while they have no adverse effect on the human umbilical vein endothelial cells (HUVECs) at the same concentration (10 μg/mL for 24 h). Hence, the authors claimed that the LY-BGs could be potentially used for anticancer applications, too. 

Other anticancer approaches involving the use of functionalized BGs include the surface grafting of chemotherapeutic drugs in combination with specific targeting molecules, such as folic acid. For example, Lin et al. [92] could functionalize MBGs using folic acid (FA) to prepare a localized site-specific anticancer delivery system. The authors evaluated the release profile of hydrophobic anticancer drug camptothecin (CPT) from the functionalized samples and observed a sustained-release trend for it. The in vitro study showed that cellular uptakes of MBG-FA were considerably higher in HeLa epithelial carcinoma cells as compared to non-cancerous fibroblasts, due to the overexpression of folate receptors (FRs) in cancer cells.

A ferrimagnetic bioactive glass-ceramic (SC45), containing magnetite as a crystalline phase, has been functionalized with chemotherapeutic drugs (doxorubicin and cisplatinum) in order to combine hyperthermic therapy and chemotherapy [93]. Both antineoplastic agents were able to bind with superficial hydroxyls exposed groups and revealed different kinetics of uptake and release. Moreover, the same glass-ceramic has been grafted with gallic acid in order to explore the opportunity to obtain anticancer activity with more natural and less toxic compounds [62]. In a similar way, natural polyphenols grafted to the surface of bioactive glass demonstrated the ability to promote the growth of healthy osteoblast cell and to selectively kill cancerous one [94].

Natural polyphenols (from sage, grape shins or tea leaves) were also considered to impart antioxidant properties to bioactive glasses [95,96]. As far as sage polyphenols are concerned, they were introduced during the bioactive glass sol-gel synthesis, while in the case of grape and tea polyphenols they were grafted to the surface of a bioactive glass without the employment of any coupling agent. Surface functionalization of bioactive glasses with natural compounds is gaining increasing interest and is the topic of a recent review in the field [97]. Surface functionalized BGs were also proposed for DNA delivery in medical and biotechnological applications [98].

## 4. Concluding Remarks

Surface functionalization is a versatile tool for the modification of the outermost surface layer of biomaterials. In this way, it is possible to tailor the interaction of materials with the biological environment, which mainly occurs at the interface, without altering material bulk properties and allowing the combination of the starting features of the specific materials with the ones added at the surface. This kind of procedure is widely studied in the field of polymeric and metallic materials, but is relatively poorly explored in the case of BGs.

BGs are known because of their peculiar reactivity and ability to chemically bond to bone and soft tissues, as well as their ability to release specific ions able to modulate the biological response. These extremely interesting properties can initially induce a certain skepticism about the surface modification of these materials: What will happen to bioactivity and ion release upon functionalization? The scientific literature can encouragingly answer to this question: In fact, the research works published so far (and here reviewed) on surface functionalization of BGs confirm that bioactivity is generally maintained after functionalization and, in addition, the glass is enriched with new specific properties, thus, generating multifunctional materials with high added value.

Moreover, surface reactivity of BGs can be effectively employed for the development of grafting procedures. Considering at these results, surface functionalization of BGs seems to be a highly promising opportunity to further tailor glass properties for the development of innovative and versatile smart implantable platforms for customized therapeutic applications.

## Figures and Tables

**Table 1:** A summary of previously performed studies with the aim of surface modification and functionalization of BGs.

Glass/Composition	Agent	Techniques	Remarks	Ref (s)
45S5 BG (46.1SiO_2_-24.4Na_2_O-26.9CaO-2.6P_2_O_5_ mol.%)	Alkaline phosphatase (ALP)	Immersed in solution	Increased enzymatic activity	[40,41,42]
45S5 BG (46.1SiO_2_-24.4Na_2_O-26.9CaO-2.6P_2_O_5_ mol.%)	(3-aminopropyl) triethoxysilane (APTS)	Immersed in solution	Improving the mechanical stability Promoting the formation of spherical HCA layer on the surface of BGs Increasing the kinetics of the release of collagen Increasing the surface roughness	[43]
45S5 BG (46.1SiO_2_-24.4Na_2_O-26.9CaO-2.6P_2_O_5_ mol.%)	APTS/GA	Immersed in solution	Enhancing the protein adsorption and collagen release Reducing the aggressiveness of the adsorption process of proteins Improving the stability of the protein attachment onto BGs	[44,45]
45Ca30 BG (45.7SiO_2_-24.1Na_2_O-26.6CaO-2.6P_2_O_5_-1.0Ga_2_O_3_ mol.%)	Tetraethoxysilane (TEOS)	Immersed in solution	Facilitating the formation of silica layer with negativity charge around BGs Increasing the surface roughness	[46]
45S5 BG (46.1SiO_2_-24.4Na_2_O-26.9CaO-2.6P_2_O_5_ mol.%)	-	plasma	A rapid and clean method	[47]
45S5 BG (46.1SiO_2_-24.4Na_2_O-26.9CaO-2.6P_2_O_5_ mol.%)	-	Core-shell based system	Accelerating HCA layer formation	[48]
Phosphate glass-ceramics (CaO-P_2_O_5_-Na_2_O-SrO-ZnO)	HF, HCl, NH_3_	Immersed in solutions with various pH values	Showing critical role of pH on the surface morphology of the materials	[49]
Mesoporous 58S BG (60SiO_2_-36CaO-4P_2_O_5_ mol.%)	KH550	Immersed in solution	Improving the loading Enhancing the antibacterial effect	[50]

**Table 2:** A summary of functional groups used to graft on the glasses and the subsequent biological improvements.

Functional Group	Method of Introduction	Function	Ref (s)
SH	2-mercapto-1 ethanol or 6 mercapto-1hexanol grafting	Improve further protein and drug grafting	[54,55]
COOH/OH	Triethoxysilylpropyl succinic anhydride or tetraethoxysilane grafting	Improve further protein and drug grafting	[56,57]
NH_2_	3-aminopropyl-triethoxysilane grafting (most used), 2-amino-1 ethanol, 6 amino-1 hexanol, 2 ethanolamine or Cysteamine grafting (occasionally reported)		[24,40,41,42,46,54,55,56,57,58,59,60,61,62,63,64,65]

## References

[1] Kargozar S., Mozafari M., Hamzehlou S., Baino F. (2019). Using Bioactive Glasses in the Management of Burns. Front. Bioeng. Biotechnol..

[2] Kargozar S., Mozafari M., Hamzehlou S., Kim H.-W., Baino F. (2019). Mesoporous bioactive glasses (MBGs) in cancer therapy: Full of hope and promise. Mater. Lett..

[3] Miola M., Pakzad Y., Banijamali S., Kargozar S., Vitale-Brovarone C., Yazdanpanah A., Bretcanu O., Ramedani A., Vernè E., Mozafari M. (2019). Glass-ceramics for cancer treatment: So close, or yet so far?. Acta Biomater..

[4] Fu Q., Rahaman M., Bal B., Bonewald L., Kuroki K., Brown R. (2010). Bioactive glass scaffolds with controllable degradation rates for bone tissue engineering applications, II: In vitro and in vivo biological evaluation. J. Biomed. Mater. Res. Part A.

[5] Wu C., Fan W., Zhu Y., Gelinsky M., Chang J., Cuniberti G., Albrecht V., Friis T., Xiao Y. (2011). Multifunctional magnetic mesoporous bioactive glass scaffolds with a hierarchical pore structure. Acta Biomater..

[6] Bi L., Rahaman M.N., Day D.E., Brown Z., Samujh C., Liu X., Mohammadkhah A., Dusevich V., Eick J.D., Bonewald L.F. (2013). Effect of bioactive borate glass microstructure on bone regeneration, angiogenesis, and hydroxyapatite conversion in a rat calvarial defect model. Acta Biomater..

[7] Wu C., Fan W., Chang J. (2013). Functional mesoporous bioactive glass nanospheres: Synthesis, high loading efficiency, controllable delivery of doxorubicin and inhibitory effect on bone cancer cells. J. Mater. Chem. B.

[8] Kargozar S., Montazerian M., Hamzehlou S., Kim H.-W., Baino F. (2018). Mesoporous bioactive glasses: Promising platforms for antibacterial strategies. Acta Biomater..

[9] Kargozar S., Baino F., Hamzehlou S., Hill R.G., Mozafari M. (2018). Bioactive glasses: Sprouting angiogenesis in tissue engineering. Trends Biotechnol..

[10] Kargozar S., Baino F., Hamzehlou S., Hill R.G., Mozafari M. (2018). Bioactive glasses entering the mainstream. Drug Discov. Today.

[11] Oyane A., Onuma K., Ito A., Kim H.M., Kokubo T., Nakamura T. (2003). Formation and growth of clusters in conventional and new kinds of simulated body fluids. J. Biomed. Mater. Res. Part A Off. J. Soc. Biomater. Jpn. Soc. Biomater. Aust. Soc. Biomater. Korean Soc. Biomater..

[12] Kokubo T., Takadama H. (2006). How useful is SBF in predicting in vivo bone bioactivity?. Biomaterials.

[13] Bohner M., Lemaitre J. (2009). Can bioactivity be tested in vitro with SBF solution?. Biomaterials.

[14] Dorozhkin S.V., Dorozhkina E.I. (2007). Crystallization from a milk-based revised simulated body fluid. Biomed. Mater..

[15] Macon A.L., Kim T.B., Valliant E.M., Goetschius K., Brow R.K., Day D.E., Hoppe A., Boccaccini A.R., Kim I.Y., Ohtsuki C. (2015). A unified in vitro evaluation for apatite-forming ability of bioactive glasses and their variants. J. Mater. Sci. Mater. Med..

[16] Mozafari M., Banijamali S., Baino F., Kargozar S., Hill R.G. (2019). Calcium carbonate: Adored and ignored in bioactivity assessment. Acta Biomater..

[17] Hench L.L. (1991). Bioceramics: From concept to clinic. J. Am. Ceram. Soc..

[18] Rehman I., Hench L., Bonfield W., Smith R. (1994). Analysis of surface layers on bioactive glasses. Biomaterials.

[19] Zhang Y., Chen L., Shi M., Zhai D., Zhu H., Chang J., Wu C., Zheng X., Yin J. (2016). Mesoporous bioactive glass nanolayer-modified zirconia coatings on Ti-6Al-4V with improved in vitro bioactivity. Int. J. Appl. Glass Sci..

[20] Omar S.A., Ballarre J., Ceré S. (2016). Protection and functionalization of AISI 316 L stainless steel for orthopedic implants: Hybrid coating and sol gel glasses by spray to promote bioactivity. Electrochim. Acta.

[21] Kuo P.H., Joshi S.S., Lu X., Ho Y.H., Xiang Y., Dahotre N.B., Du J. (2019). Laser coating of bioactive glasses on bioimplant titanium alloys. Int. J. Appl. Glass Sci..

[22] Baino F., Verné E. (2017). Glass-based coatings on biomedical implants: A state-of-the-art review. Biomed. Glasses.

[23] Duan K., Wang R. (2006). Surface modifications of bone implants through wet chemistry. J. Mater. Chem..

[24] Gruian C., Vanea E., Simon S., Simon V. (2012). FTIR and XPS studies of protein adsorption onto functionalized bioactive glass. Biochim. Biophys. Acta (BBA)-Proteins Proteomics.

[25] Filippini P., Rainaldi G., Ferrante A., Mecheri B., Gabrielli G., Bombace M., Indovina P.L., Santini M.T. (2001). Modulation of osteosarcoma cell growth and differentiation by silane-modified surfaces. J. Biomed. Mater. Res..

[26] Ferraris S., Vernè E. (2016). Surface Functionalization of Bioactive Glasses: Reactive Groups, Biomolecules and Drugs on Bioactive Surfaces for Smart and Functional Biomaterials. Bioactive Glasses: Fundamental, Technology and Applications.

[27] Baino F., Ferraris S., Miola M., Verné E., Evans I., Bretcanu O. (2019). Multifunctional Bioactive Glasses and Glass-Ceramics: Beyond ‘Traditional’ Bioactivity. Biomedical, Therapeutic and Clinical Applications of Bioactive Glasses.

[28] Chang J., Zhou Y. (2017). Surface modification of bioactive glasses. Bioactive Glasses.

[29] Wu C., Zhang Y., Ke X., Xie Y., Zhu H., Crawford R., Xiao Y. (2010). Bioactive mesopore-glass microspheres with controllable protein-delivery properties by biomimetic surface modification. J. Biomed. Mater. Res. Part A.

[30] Lee W.-H., Loo C.-Y., Rohanizadeh R. (2014). A review of chemical surface modification of bioceramics: Effects on protein adsorption and cellular response. Colloids Surf. B Biointerfaces.

[31] Spriano S., Yamaguchi S., Baino F., Ferraris S. (2018). A critical review of multifunctional titanium surfaces: New frontiers for improving osseointegration and host response, avoiding bacteria contamination. Acta Biomater..

[32] Izquierdo-Barba I., Vallet-Regí M. (2015). Mesoporous bioactive glasses: Relevance of their porous structure compared to that of classical bioglasses. Biomed. Glasses.

[33] Wu C., Chang J. (2012). Mesoporous bioactive glasses: Structure characteristics, drug/growth factor delivery and bone regeneration application. Interface Focus.

[34] Baino F., Fiume E., Miola M., Verné E. (2018). Bioactive sol-gel glasses: Processing, properties, and applications. Int. J. Appl. Ceram. Technol..

[35] Baino F., Fiume E., Barberi J., Kargozar S., Marchi J., Massera J., Verné E. (2019). Processing methods for making porous bioactive glass-based scaffolds—A state-of-the-art review. Int. J. Appl. Ceram. Technol..

[36] Baino F. (2018). Bioactive glasses—When glass science and technology meet regenerative medicine. Ceram. Int..

[37] Kargozar S., Hamzehlou S., Baino F. (2019). Can bioactive glasses be useful to accelerate the healing of epithelial tissues?. Mater. Sci. Eng. C.

[38] Baino F., Hamzehlou S., Kargozar S. (2018). Bioactive Glasses: Where Are We and Where Are We Going?. J. Funct. Biomater..

[39] Kargozar S., Hamzehlou S., Baino F. (2017). Potential of Bioactive Glasses for Cardiac and Pulmonary Tissue Engineering. Materials.

[40] Verné E., Vitale-Brovarone C., Bui E., Bianchi C., Boccaccini A. (2009). Surface functionalization of bioactive glasses. J. Biomed. Mater. Res. Part A Off. J. Soc. Biomater. Jpn. Soc. Biomater. Aust. Soc. Biomater. Korean Soc. Biomater..

[41] Verné E., Ferraris S., Vitale-Brovarone C., Cochis A., Rimondini L. (2014). Bioactive glass functionalized with alkaline phosphatase stimulates bone extracellular matrix deposition and calcification in vitro. Appl. Surf. Sci..

[42] Verné E., Ferraris S., Cassinelli C., Boccaccini A. (2015). Surface functionalization of Bioglass^®^ with alkaline phosphatase. Surf. Coat. Technol..

[43] Hum J., Boccaccini A. (2018). Collagen as coating material for 45S5 bioactive glass-based scaffolds for bone tissue engineering. Int. J. Mol. Sci..

[44] Chen Q., Rezwan K., Armitage D., Nazhat S., Boccaccini A. (2006). The surface functionalization of 45S5 Bioglass^®^-based glass-ceramic scaffolds and its impact on bioactivity. J. Mater. Sci. Mater. Med..

[45] Gruian C., Boehme S., Simon S., Steinhoff H.-J., Klare J. (2014). Assembly and function of the tRNA-modifying GTPase MnmE adsorbed to surface functionalized bioactive glass. ACS Appl. Mater. Interfaces.

[46] Lusvardi G., Malavasi G., Menabue L., Shruti S. (2013). Gallium-containing phosphosilicate glasses: Functionalization and in-vitro bioactivity. Mater. Sci. Eng. C.

[47] Larranaga A., Petisco S., Sarasua J. (2013). Improvement of thermal stability and mechanical properties of medical polyester composites by plasma surface modification of the bioactive glass particles. Polym. Degrad. Stab..

[48] Lopes J.H., Fonseca E.M.B., Mazali I.O., Magalhães A., Landers R., Bertran C.A. (2017). Facile and innovative method for bioglass surface modification: Optimization studies. Mater. Sci. Eng. C.

[49] Li X., Cai S., Zhang W., Xu G., Zhou W. (2009). Effect of pH values on surface modification and solubility of phosphate bioglass-ceramics in the CaO–P_2_O_5_–Na_2_O–SrO–ZnO system. Appl. Surf. Sci..

[50] Zhu H., Hu C., Zhang F., Feng X., Li J., Liu T., Chen J., Zhang J. (2014). Preparation and antibacterial property of silver-containing mesoporous 58S bioactive glass. Mater. Sci. Eng. C.

[51] Mishra M., Muthuprasanna P., Prabha K.S., Rani P.S., Babu I.A.S., Chandiran I.S., Arunachalam G., Shalini S. (2009). Basics and potential applications of surfactants—A review. Int. J. PharmTech Res..

[52] Sagnella S., Mai-Ngam K. (2005). Chitosan based surfactant polymers designed to improve blood compatibility on biomaterials. Colloids Surf. B Biointerfaces.

[53] Mitchell M.J., Castellanos C.A., King M.R. (2015). Surfactant functionalization induces robust, differential adhesion of tumor cells and blood cells to charged nanotube-coated biomaterials under flow. Biomaterials.

[54] Bonici A., Lusvardi G., Malavasi G., Menabue L., Piva A. (2012). Synthesis and characterization of bioactive glasses functionalized with Cu nanoparticles and organic molecules. J. Eur. Ceram. Soc..

[55] Aina V., Marchis T., Laurenti E., Diana E., Lusvardi G., Malavasi G., Menabue L., Cerrato G., Morterra C. (2010). Functionalization of sol gel bioactive glasses carrying Au nanoparticles: Selective Au affinity for amino and thiol ligand groups. Langmuir.

[56] Sun J., Li Y., Li L., Zhao W., Li L., Gao J., Ruan M., Shi J. (2008). Functionalization and bioactivity in vitro of mesoporous bioactive glasses. J. Non-Cryst. Solids.

[57] Chen Q.-Z., Rezwan K., Françon V., Armitage D., Nazhat S.N., Jones F.H., Boccaccini A.R. (2007). Surface functionalization of Bioglass^®^-derived porous scaffolds. Acta Biomater..

[58] El-Fiqi A., Lee J.H., Lee E.-J., Kim H.-W. (2013). Collagen hydrogels incorporated with surface-aminated mesoporous nanobioactive glass: Improvement of physicochemical stability and mechanical properties is effective for hard tissue engineering. Acta Biomater..

[59] Manzano M., Aina V., Arean C., Balas F., Cauda V., Colilla M., Delgado M., Vallet-Regi M. (2008). Studies on MCM-41 mesoporous silica for drug delivery: Effect of particle morphology and amine functionalization. Chem. Eng. J..

[60] Aina V., Malavasi G., Magistris C., Cerrato G., Martra G., Viscardi G., Menabue L., Lusvardi G. (2014). Conjugation of amino-bioactive glasses with 5-aminofluorescein as probe molecule for the development of pH sensitive stimuli-responsive biomaterials. J. Mater. Sci. Mater. Med..

[61] Aina V., Ghigo D., Marchis T., Cerrato G., Laurenti E., Morterra C., Malavasi G., Lusvardi G., Menabue L., Bergandi L. (2011). Novel bio-conjugate materials: Soybean peroxidase immobilized on bioactive glasses containing Au nanoparticles. J. Mater. Chem..

[62] Schickle K., Zurlinden K., Bergmann C., Lindner M., Kirsten A., Laub M., Telle R., Jennissen H., Fischer H. (2011). Synthesis of novel tricalcium phosphate-bioactive glass composite and functionalization with rhBMP-2. J. Mater. Sci. Mater. Med..

[63] Chen Q., Ahmed I., Knowles J., Nazhat S., Boccaccini A., Rezwan K. (2008). Collagen release kinetics of surface functionalized 45S5 Bioglass^®^-based porous scaffolds. J. Biomed. Mater. Res. Part A Off. J. Soc. Biomater. Jpn. Soc. Biomater. Aust. Soc. Biomater. Korean Soc. Biomater..

[64] Verné E., Ferraris S., Vitale-Brovarone C., Spriano S., Bianchi C.L., Naldoni A., Morra M., Cassinelli C. (2010). Alkaline phosphatase grafting on bioactive glasses and glass ceramics. Acta Biomater..

[65] Ferraris S., Vitale-Brovarone C., Bretcanu O., Cassinelli C., Vernè E. (2013). Surface functionalization of 3D glass—Ceramic porous scaffolds for enhanced mineralization in vitro. Appl. Surf. Sci..

[66] (2015). adhesion of tumor cells and blood cells to charged nanotube-coated biomaterials under flow. Biomaterials.

[67] Wang S., Wen S., Shen M., Guo R., Cao X., Wang J., Shi X. (2011). Aminopropyltriethoxysilane-mediated surface functionalization of hydroxyapatite nanoparticles: Synthesis, characterization, and in vitro toxicity assay. Int. J. Nanomed..

[68] Magyari K., Baia L., Vulpoi A., Simon S., Popescu O., Simon V. (2015). Bioactivity evolution of the surface functionalized bioactive glasses. J. Biomed. Mater. Res. Part B Appl. Biomater..

[69] Chen X., Guo C., Zhao N. (2008). Preparation and characterization of the sol—Gel nano-bioactive glasses modified by the coupling agent gamma—Aminopropyltriethoxysilane. Appl. Surf. Sci..

[70] Curran J.M., Chen R., Hunt J.A. (2005). Controlling the phenotype and function of mesenchymal stem cells in vitro by adhesion to silane-modified clean glass surfaces. Biomaterials.

[71] Stanić V. (2017). Variation in properties of bioactive glasses after surface modification. Clinical Applications of Biomaterials.

[72] Massera J., Mishra A., Guastella S., Ferraris S., Verné E. (2016). Surface functionalization of phosphate-based bioactive glasses with 3-aminopropyltriethoxysilane (APTS). Biomed. Glasses.

[73] Ferraris S., Nommeots-Nomm A., Spriano S., Vernè E., Massera J. (2019). Surface reactivity and silanization ability of borosilicate and Mg-Sr-based bioactive glasses. Appl. Surf. Sci..

[74] Ozmen M., Can K., Akin I., Arslan G., Tor A., Cengeloglu Y., Ersoz M. (2009). Surface modification of glass beads with glutaraldehyde: Characterization and their adsorption property for metal ions. J. Hazard. Mater..

[75] Gruian C., Vulpoi A., Steinhoff H.-J., Simon S. (2012). Structural changes of methemoglobin after adsorption on bioactive glass, as a function of surface functionalization and salt concentration. J. Mol. Struct..

[76] Leivo J., Virjula S., Vanhatupa S., Kartasalo K., Kreutzer J., Miettinen S., Kallio P. (2017). A durable and biocompatible ascorbic acid-based covalent coating method of polydimethylsiloxane for dynamic cell culture. J. R. Soc. Interface.

[77] Gruian C., Vulpoi A., Vanea E., Oprea B., Steinhoff H.-J., Simon S. (2013). The attachment affinity of hemoglobin toward silver-containing bioactive glass functionalized with glutaraldehyde. J. Phys. Chem. B.

[78] Wiącek A.E., Gozdecka A., Jurak M., Przykaza K., Terpiłowski K. (2018). Wettability of plasma modified glass surface with bioglass layer in polysaccharide solution. Colloids Surf. A Physicochem. Eng. Asp..

[79] Araújo M., Viveiros R., Philippart A., Miola M., Doumett S., Baldi G., Perez J., Boccaccini A.R., Aguiar-Ricardo A., Verné E. (2017). Bioactivity, mechanical properties and drug delivery ability of bioactive glass-ceramic scaffolds coated with a natural-derived polymer. Mater. Sci. Eng. C.

[80] Srdić V.V., Mojić B., Nikolić M., Ognjanović S. (2013). Recent progress on synthesis of ceramics core/shell nanostructures. Matrix.

[81] Verné E., Ferraris S., Miola M., Fucale G., Maina G., Martinasso G., Canuto R.A., Di Nunzio S., Vitale-Brovarone C. (2008). Synthesis and characterisation of bioactive and antibacterial glass–ceramic Part 1–Microstructure, properties and biological behaviour. Adv. Appl. Ceram..

[82] Verné E., Miola M., Brovarone C.V., Cannas M., Gatti S., Fucale G., Maina G., Massé A., Di Nunzio S. (2009). Surface silver-doping of biocompatible glass to induce antibacterial properties. Part I: Massive glass. J. Mater. Sci. Mater. Med..

[83] Vernè E., Di Nunzio S., Bosetti M., Appendino P., Brovarone C.V., Maina G., Cannas M. (2005). Surface characterization of silver-doped bioactive glass. Biomaterials.

[84] Miola M., Ferraris S., Di Nunzio S., Robotti P., Bianchi G., Fucale G., Maina G., Cannas M., Gatti S., Massé A. (2009). Surface silver-doping of biocompatible glasses to induce antibacterial properties. Part II: Plasma sprayed glass-coatings. J. Mater. Sci. Mater. Med..

[85] Verné E., Ferraris S., Miola M., Fucale G., Maina G., Robotti P., Bianchi G., Martinasso G., Canuto R.A., Vitale-Brovarone C. (2008). Synthesis and characterisation of bioactive and antibacterial glass-ceramic Part 2—Plasma spray coatings on metallic substrates. Adv. Appl. Ceram..

[86] Farag M., Abd-Allah W., Ahmed H.Y. (2017). Study of the dual effect of gamma irradiation and strontium substitution on bioactivity, cytotoxicity, and antimicrobial properties of 45S5 bioglass. J. Biomed. Mater. Res. Part A.

[87] Shaikh S., Kedia S., Singh A.K., Sharma K., Sinha S. (2017). Surface treatment of 45S5 Bioglass using femtosecond laser to achieve superior growth of hydroxyapatite. J. Laser Appl..

[88] Pou P., Riveiro A., del Val J., Comesaña R., Penide J., Arias-González F., Soto R., Lusquiños F., Pou J. (2017). Laser surface texturing of Titanium for bioengineering applications. Procedia Manuf..

[89] Zhao S., Zhang J., Zhu M., Zhang Y., Liu Z., Ma Y., Zhu Y., Zhang C. (2015). Effects of functional groups on the structure, physicochemical and biological properties of mesoporous bioactive glass scaffolds. J. Mater. Chem. B.

[90] Shaikh S., Singh D., Subramanian M., Kedia S., Singh A.K., Singh K., Gupta N., Sinha S. (2018). Femtosecond laser induced surface modification for prevention of bacterial adhesion on 45S5 bioactive glass. J. Non-Cryst. Solids.

[91] Zheng K., Lu M., Liu Y., Chen Q., Taccardi N., Hüser N., Boccaccini A.R. (2016). Monodispersed lysozyme-functionalized bioactive glass nanoparticles with antibacterial and anticancer activities. Biomed. Mater..

[92] Lin H.-M., Lin H.-Y., Chan M.-H. (2013). Preparation, characterization, and in vitro evaluation of folate-modified mesoporous bioactive glass for targeted anticancer drug carriers. J. Mater. Chem. B.

[93] Vernè E., Miola M., Ferraris S., Bianchi C.L., Naldoni A., Maina G., Bretcanu O. (2010). Surface activation of a ferrimagnetic glass—Ceramic for antineoplastic drugs grafting. Adv. Eng. Mater..

[94] Cazzola M., Verné E., Cochis A., Sorrentino R., Azzimonti B., Prenesti E., Rimondini L., Ferraris S. (2017). Bioactive glasses functionalized with polyphenols: In vitro interactions with healthy and cancerous osteoblast cells. J. Mater. Sci..

[95] Cazzola M., Corazzari I., Prenesti E., Bertone E., Vernè E., Ferraris S. (2016). Bioactive glass coupling with natural polyphenols: Surface modification, bioactivity and anti-oxidant ability. Appl. Surf. Sci..

[96] Dziadek M., Dziadek K., Zagrajczuk B., Menaszek E., Cholewa-Kowalska K. (2016). Poly (ε-caprolactone)/bioactive glass composites enriched with polyphenols extracted from sage (*Salvia officinalis* L.). Mater. Lett..

[97] Schuhladen K., Roether J.A., Boccaccini A.R. (2019). Bioactive glasses meet phytotherapeutics: The potential of natural herbal medicines to extend the functionality of bioactive glasses. Biomaterials.

[98] Li X., Zhao L., Liang Q., Ye J., Komatsu N., Zhang Q., Gao W., Xu M., Chen X. (2017). Cationic polyarginine conjugated mesoporous bioactive glass nanoparticles with polyglycerol coating for efficient DNA delivery. J. Biomed. Nanotechnol..

